# Plasma extracellular vesicle messenger RNA profiling identifies prognostic EV signature for non-invasive risk stratification for survival prediction of patients with pancreatic ductal adenocarcinoma

**DOI:** 10.1186/s13045-023-01404-w

**Published:** 2023-02-03

**Authors:** Yi Han, Pascal Drobisch, Alexander Krüger, Doreen William, Konrad Grützmann, Lukas Böthig, Heike Polster, Lena Seifert, Adrian M. Seifert, Marius Distler, Mathieu Pecqueux, Carina Riediger, Verena Plodeck, Heiner Nebelung, Georg F. Weber, Christian Pilarsky, Ulf Kahlert, Ulf Hinz, Susanne Roth, Thilo Hackert, Jürgen Weitz, Fang Cheng Wong, Christoph Kahlert

**Affiliations:** 1 Department of Visceral, Thoracic and Vascular Surgery, University Hospital and Faculty of Medicine Carl Gustav Carus, Technische Universität Dresden, Fetscherstr. 74, 01307 Dresden, Germany; 2 Core Unit for Molecular Tumour Diagnostics, National Center for Tumour Diseases, Dresden, Germany; 3 German Cancer Consortium (DKTK), German Cancer Research Centre (DKFZ), Heidelberg, Germany; 4 National Center for Tumour Diseases, Partner Site Dresden, Heidelberg, Germany; 5 Department of Diagnostic and Interventional Radiology, Carl Gustav Carus University Hospital Dresden, Dresden, Germany; 6 Department of Surgery, Universitätsklinikum Erlangen, Erlangen, Germany; 7 Molecular and Experimental Surgery, University Clinic for General-, Visceral-, Vascular- and Transplantation Surgery, Medical Faculty University Hospital Magdeburg, Magdeburg, Germany; 8 Department of General, Visceral and Transplantation Surgery, Heidelberg University Hospital, Heidelberg, Germany

**Keywords:** Extracellular vesicles, Pancreatic ductal adenocarcinoma, Prognosis, Plasma extracellular vesicle, mRNA biomarkers

## Abstract

**Background:**

The prognosis of pancreatic ductal adenocarcinoma (PDAC) is one of the most dismal of all cancers and the median survival of PDAC patients is only 6–8 months after diagnosis. While decades of research effort have been focused on early diagnosis and understanding of molecular mechanisms, few clinically useful markers have been universally applied. To improve the treatment and management of PDAC, it is equally relevant to identify prognostic factors for optimal therapeutic decision-making and patient survival. Compelling evidence have suggested the potential use of extracellular vesicles (EVs) as non-invasive biomarkers for PDAC. The aim of this study was thus to identify non-invasive plasma-based EV biomarkers for the prediction of PDAC patient survival after surgery.

**Methods:**

Plasma EVs were isolated from a total of 258 PDAC patients divided into three independent cohorts (discovery, training and validation). RNA sequencing was first employed to identify differentially-expressed EV mRNA candidates from the discovery cohort (*n* = 65) by DESeq2 tool. The candidates were tested in a training cohort (*n* = 91) by digital droplet polymerase chain reaction (ddPCR). Cox regression models and Kaplan–Meier analyses were used to build an EV signature which was subsequently validated on a multicenter cohort (*n* = 83) by ddPCR.

**Results:**

Transcriptomic profiling of plasma EVs revealed differentially-expressed mRNAs between long-term and short-term PDAC survivors, which led to 10 of the top-ranked candidate EV mRNAs being tested on an independent training cohort with ddPCR. The results of ddPCR enabled an establishment of a novel prognostic EV mRNA signature consisting of *PPP1R12A*, *SCN7A* and *SGCD* for risk stratification of PDAC patients. Based on the EV mRNA signature, PDAC patients with high risk displayed reduced overall survival (OS) rates compared to those with low risk in the training cohort (*p* = 0.014), which was successfully validated on another independent cohort (*p* = 0.024). Interestingly, the combination of our signature and tumour stage yielded a superior prognostic performance (*p* = 0.008) over the signature (*p* = 0.022) or tumour stage (*p* = 0.016) alone. It is noteworthy that the EV mRNA signature was demonstrated to be an independent unfavourable predictor for PDAC prognosis.

**Conclusion:**

This study provides a novel and non-invasive prognostic EV mRNA signature for risk stratification and survival prediction of PDAC patients.

**Supplementary Information:**

The online version contains supplementary material available at 10.1186/s13045-023-01404-w.

## Background

Pancreatic ductal adenocarcinoma (PDAC) constitutes approximately 90% of pancreatic cancer, which is widely recognized as one of the most deadly cancers in men and women with an extremely high mortality rate worldwide [1]. Patients with PDAC have only a median survival rate of 6–8 months after diagnosis and the 5-year overall survival rate remains as low as 11% [2, 3]. Therapeutic advances and prognostic information is tempered by the fact that the nature of PDAC is heterogenous at molecular, pathological and clinical levels, which renders limited opportunities for survival improvement [4]. For instance, genomic signatures, molecular subtypes arising from large-scale transcriptomic profiling, histological subtypes from World Health Organization (WHO) classification and the pattern of disease progression have been attempted to be used for therapeutic and prognostic applications, but the outcome has been less than satisfactory [5–7]. Hence, there still remains a significant unmet clinical need for the discovery of specific and sensitive, yet non-invasive, biomarkers for survival prediction of PDAC patients in order to optimize therapeutic stratification and management.

In a search for such biomarkers, circulating extracellular vesicles (EVs) have come into the spotlight of research as a high potential source of liquid biopsy. It is particularly attractive in the context of PDAC because sampling of the primary tumour itself is an invasive method with risk of infection and spreading of tumour cells. Numerous studies have shown the potential of EV cargoes as potential biomarkers for PDAC clinical applications. For instance, a higher percentage of PDAC patients exhibited detectable mutant KRAS in their circulating EV-derived DNAs compared to age-matched healthy controls and this is associated with decreased disease-free survival rates in patients with localized disease, suggesting the diagnostic and prognostic potential of EV-derived DNAs [8]. Apart from that, a panel of protein cargoes (e.g., BAIAP2L1, ALPL, PTPRJ, FCER1G and TMEM2) in circulating plasma EVs, or single serum-derived EV protein Glypican-1 and/or in combination with other protein markers EGFR, EpCAM, MUC1 and WNT2 from PDAC patients have been described as biomarkers for early cancer detection with high accuracy and sensitivity [9–11]. As for RNAs, majority of the hitherto studies focused on microRNAs (miRNAs), long RNAs (mainly messenger RNA, long non-coding RNA and circular RNA) and transfer RNAs (tRNAs) as diagnostic and prognostic cancer biomarkers [12–14]. In particular, a panel of long RNAs (FGA, KRT19, HIST1H2BK, ITIH2, MARCH2, CLDN1, MAL2 and TIMP1) from plasma EVs have been successfully validated for early diagnosis of PDAC with an area under the curve (AUC) of 0.936, indicating the potential of long RNAs from EVs as diagnostic factors that is superior to the current diagnostic marker for PDAC, carbohydrate antigen 19-9 (CA19-9) [12]. Despite all the promising results from the fast-growing EV research field, it is noticeable that the potential role of mRNAs from the circulating EVs in prognostic application for PDAC has been largely unexplored.

The aim of this study was to identify non-invasive EV mRNA biomarkers for predicting the survival of PDAC patients after surgery. It was hypothesized that plasma EVs contained mRNA cargoes that could be employed as prognostic biomarkers for PDAC patient survival prediction. By examining plasma EV transcriptomes from long-term and short-term PDAC survivors in a discovery cohort and testing the selected candidates on another training cohort of PDAC patients, a prognostic EV 3-mRNA signature was identified in this study. The EV signature allowed for risk stratification of PDAC patients in both the training and the validation cohorts, with the high-risk group displaying reduced overall survival (OS) rates compared to the low-risk group. Importantly, this EV 3-mRNA signature revealed a superior prognostic performance when combined with tumour stage, suggesting that it can act as a complement to the current prognostic factor. Moreover, univariate and multivariate analysis showed that the EV 3-mRNA signature is an independent unfavourable predictor for PDAC prognosis. These data suggest that plasma EV mRNAs can serve as biomarkers for predicting OS of PDAC patients by non-invasive risk stratification. The fundamental goal of this study is to fill up the current EV research gap by providing an insight into the prognosis of PDAC with plasma EV mRNAs, highlighting an emerging area in which there is much to explore.

## Methods

### Patient samples and clinical characteristics

This investigation involving human samples was designed as a retrospective exploratory study and complied to most of the Reporting Recommendations for Tumour Marker Prognostic Studies (REMARK) criteria [15]. A total of 239 PDAC patients with stage I-IV were included in this study based on the histopathology confirmation and selection criterion (Additional file 1: Fig. S1). Plasma samples from the enrolled patients were received from the (1) Department of Visceral, Thoracic and Vascular Surgery, University Hospital Carl Gustav Carus Dresden (UHD), Dresden, Germany; (2) Department of General, Visceral and Transplantation Surgery, University Hospital Heidelberg (UHH), Heidelberg, Germany; and (3) Department of Surgery, University Hospital Erlangen (UHE), Erlangen, Germany after approval by the local Institutional Review Board/ethics committee (Dresden: EK76032013; Heidelberg: 159/2002; Erlangen: 156_19B). Written informed consent from the patients was obtained pre-operatively with the disclosure of research purpose.

As there were no previous studies investigating the prognostic relevance of EVs in patients with PDAC, the study started with a discovery cohort comprising 65 PDAC patients from UHD intentionally divided into long-term survivors (OS of longer than 36 months, *n* = 39) and short-term survivors (OS between 6–12 months, *n* = 26) for identification of potential prognostic EV biomarkers from the two extreme spectrums of the survival rates of PDAC patients. Hence, the inclusion criterion for this discovery cohort were: (1) histologically verified PDAC; (2) survival days between 6 and 12 months or longer than 36 months; and (3) patients with a minimum age of 18 years. Some factors may have an impact on the overall patient survival such as neoadjuvant therapy, T1 and M1 tumours as well as R2 resections. PDAC patients who received neoadjuvant therapy, with T1 and M1 tumours, R2 resection, survival days shorter than 6 months (185.5 days) or between 12 and 36 months (364–1095 days), missing survival data or less than 500 μl plasma volume were excluded in the discovery cohort.

Subsequently, a training cohort consisted of 91 PDAC patients from UHH was employed to evaluate the potential prognostic EV candidate biomarkers in a blinded fashion (i.e., the clinical information of the patients were obtained only after the experiments), which was followed by an independent and multi-center validation cohort made up of 83 PDAC patients (UHD = 63; UHE = 20). Clinical information included gender, age, tumour stage, tumour grade (G), resection margin, neoadjuvant therapy, adjuvant therapy, CA19-9 levels and follow-up time were summarized into Table 1. The clinical endpoint examined in this study was OS, which was defined as the time of surgery to the time of death or last follow-up. All the patients with OS shorter than 30 days were excluded to avoid cases of death due to surgical complications.

**Table 1:** The characteristics of PDAC patients for the discovery, training and validation cohorts

	Discovery cohort (UHD)		Training cohort (UHH)	Validation cohort (UHD + UHE)
	Long-term survivors	Short-term survivors		
Total number, *n*	39 (60.0%)	26 (40.0%)	91	83
Gender				
Male	19 (48.7%)	10 (38.5%)	42 (46.2%)	45 (54.2%)
Female	20 (51.3%)	16 (61.5%)	49 (53.9%)	38 (45.8%)
Unknown	0	0	0	0
Age (years)				
Median (range)	66.1 (40–79)	68.3 (45–83)	67 (33–84)	70 (43–83)
Unknown	0	0	0	0
Tumour stage				
IA	1 (2.6%)	1 (3.9%)	0	3 (3.61%)
IB	14 (35.9%)	5 (19.2%)	0	6 (7.2%)
IIA	5 (12.8%)	0	10 (11.0%)	17 (20.5%)
IIB	10 (25.6%)	8 (30.8%)	42 (46.2%)	35 (42.2%)
III	9 (23.1%)	12 (46.2%)	20 (22.0%)	7 (8.4%)
IV	0	0	17 (18.7%)	15 (18.1%)
Unknown	0	0	2 (2.2%)	0
Cancer grade				
G1	2 (5.1%)	0	0	0
G2	20 (51.3%)	11 (42.3%)	45 (49.5%)	32 (38.6%)
G3	16 (41.0%)	15 (57.7%)	29 (31.9%)	39 (47.0%)
G4*	0	0	1(1.1%)	0
Unknown	1 (2.6%)	0	16 (17.6%)	12 (14.5%)
Resection margin				
R0	33 (84.6%)	18 (69.2%)	13 (14.3%)	64 (77.1%)
R1	6 (15.4%)	8 (30.8%)	65 (71.4%)	13 (15.7%)
R2	0	0	12 (13.2%)	1 (1.2%)
Unknown	0	0	1 (1.1%)	5 (6.0%)
Neoadjuvant chemotherapy				
Yes	0	0	8 (8.8%)	13 (15.7%)
No	0	0	35 (38.5%)	51 (61.5%)
Unknown	0	0	48 (52.8%)	19 (22.9%)
Adjuvant chemotherapy				
Yes	29 (74.4%)	15 (57.7%)	42 (46.2%)	59 (71.1%)
No	9 (23.1%)	10 (38.5%)	10 (11.0%)	2 (2.4%)
Unknown	1 (2.6%)	1 (3.9%)	39 (42.9%)	22 (26.5%)
CA19-9				
Median, U/ml (range)	59.5 (0.7–3517)	312.0 (8.5–38,878)	182.6 (1.0–9024.4)	189.2 (2.0–20,752.8)
Unknown (no. of patients)	0	0	57	6
Follow-up time				
Median, days (range)	2085.0	302.5	466.0	402.0
	(1104–4575)	(194–364)	(49–3246)	(33–3803)

^*^G4: Anaplastic carcinoma

### Plasma sample collection and processing

Nine ml of blood samples from the recruited patients were collected in an ethylene diamine tetra acetic acid (EDTA)-tube (Sarstedt, Nümbrecht, Germany) on the day of operation or up to a maximum of 10 days before the operation. The blood samples were first centrifuged at 1500×*g* for 12 min at 4 °C (centrifuge break set to 0) and the plasma (upper phase) was transferred to a 15 ml Eppendorf tube for another round of centrifugation at 1500×*g* for 12 min at 4 °C (centrifuge break set to 0). The plasma samples were then aliquoted for storage at − 80 °C until use.

### EV and RNA isolation

For isolation of EV RNAs from plasma samples, a one-step protocol using exoRNeasy Serum/Plasma Midi kit (Qiagen, Hilden, Germany) was employed according to the manufacturer’s instructions. Briefly, 550 μl plasma sample was first centrifuged at 16,000×*g* for 10 min at 4 °C to remove any cell debris or insoluble materials. The supernatant was mixed with XBP buffer (1:1 dilution) and loaded onto the exoEasy spin column and centrifuged at 500×*g* for 1 min. The column was washed with 3.5 ml XWP buffer and centrifuged at 3200×*g* for 5 min. For elution, the column was transferred into a new collection tube and 700 μl Qiazol was added and followed by another round of centrifugation at 3220×*g* for 5 min. The sample was incubated for 5 min at room temperature before adding 90 μl chloroform and further incubation for 3 min. RNAs in the upper aqueous phase were transferred to a new tube after centrifugation at 12,000×*g* for 15 min at 4 °C and mixed with 2X volume of ethanol before binding onto RNeasy MiniElute spin column. The sample was washed thrice with RWT and RPE buffers and eventually eluted with 20 μl nuclease-free water.

For elution of EVs from the exoEasy spin column without RNA isolation, 200–500 μl XE buffer was used and the eluted EVs were further concentrated with Vivaspin ® 500 filtration (100,000 MWCO, Sartorius, Göttingen, Germany) before subjecting to EV characterization of transmission electron microscopy, nanoparticle tracking analysis and western blot.

### EV isolation using ultracentrifugation

500 μl plasma samples were thawed and mixed with 500 μl PBS. The diluted plasma samples were filtered with 0.2 μm filter and subjected to ultracentrifugation at 100,000×*g*, 2 h, 4 °C in a ultracentrifuge (Sorvall MX150 + micro-ultracentrifuge, Thermo Scientific, Darmstadt, Germany). The supernatant was removed and the pellet was washed once with ice-cold Phosphate Buffered Saline (PBS, Gibco, Carlsbad, California, USA) and ultracentrifuged again at 100,000×*g*, 2 h, 4 °C. The resulting pellet was resuspended 100 μl PBS and transferred to Vivaspin ® 500 filtration (100,000 MWCO, Sartorius, Göttingen, Germany) for centrifugation at 15,000×*g*, 45 min. The concentrated EVs were stored at − 20 °C until further use for EV characterization.

### EV isolation using precipitation method

550 μl plasma samples were thawed and first centrifuged at 2000×*g* for 20 min. The supernatant was subjected to another round of centrifugation at 10,000×*g* for 20 min. After the second round of centrifugation, 500 μl supernatant was mixed with 250 μl PBS, vortexed and added with 150 μl Exosome Precipitation Reagent. The mixture was incubated at room temperature for 10 min and centrifuged at 10,000×*g* for 5 min. After removing the supernatant completely, the pellet was resuspended with 500 µl PBS and concentrated with Vivaspin ® 500 filtration (100,000 MWCO, Sartorius, Göttingen, Germany) by centrifugation at 15,000×*g*, 45 min. The resulting EVs were stored at − 20 °C until further use for EV characterization.

### Transmission electron microscopy (TEM)

Fresh EVs were submitted to Electron Microscopy Facility at the Center for Molecular and Cellular Bioengineering, Technische Universität Dresden for detection by negative staining with TEM. Briefly, 10 μl sample was transferred to a grid (300 mesh) and incubated for 10 min. The grid was washed 2X with water and dried with filter paper. The sample was quickly stained with 1% uranyl acetate/water (UA) for 20 s and the UA was slowly removed with the tip of filter paper so that the dye covered nicely around the exosomes. After complete drying, imaging of EVs was performed on a 100 kv TEM (FEI Morgagni 268D) with a SIS MegaView III camera.

### Nanoparticle tracking analysis (NTA)

For quantification of the number and size distribution of EVs, the rate of Brownian motion of the particles was determined by ZetaView® nanoparticle tracking analyzer (ZetaView 8.05.05 SP2, Particle Metrix GmbH, Meerbusch, Germany). Alignment particles (Particle Metrix GmbH, Meerbusch, Germany) with a known average size of 100 nm were used to calibrate the instrument prior to sample readings. The instrument pre-acquisition parameters were set to a sensitivity of 80 and shutter of 70, whereas post-acquisition parameters were set to a minimum brightness of 20, a maximum size of 200 pixels, and a minimum size of 5 pixels. Samples were diluted in PBS to achieve a particle count in the range of 50 to 200 particles per visual field. EV samples were usually diluted in PBS with dilution factor 1:10,000 and were loaded to the instrument and analyzed based on the videos taken at 11 different positions (30 s each video) throughout the cell. After automated analysis of all 11 positions and removal of outlier position if any, the concentration, mean and median diameters of the sample were calculated by the machine software.

### Western blot

EV samples were lysed in RIPA lysis buffer (Cell Signalling Technology, Danvers, Massachusetts, United States) complemented with 100X Halt™ protease and phosphatase inhibitor single use cocktail (Thermo Scientific, Darmstadt, Germany) for at least 30 min on ice and centrifuged at 10,000×*g* for 30 min, 4 °C to remove insoluble materials. Protein concentration of the samples was determined by the Pierce™ BCA Protein Assay Kit (ThermoFischer Scientific, Rockford, USA) according to manufacturer’s instructions and measured the absorbance at 562 nm using microplate reader (Varioskan LUX Multimode Microplate Reader, Thermo Scientific, Darmstadt, Germany). BSA standard curve with a range of 0 to 2000 µg/ml was used to calculate the concentration of the samples. Cell lysates were then mixed in NuPAGE 4X LDS sample buffer dye (Invitrogen, Carlsbad, California, USA) and NuPAGE 10X sample reducing agent (Life Technologies, Carlsbad, California, USA) before boiling at 90 °C for 10 min and subjecting to SDS-PAGE polyacrylamide gel electrophoresis (4–12% Bis–Tris gel, Life Technologies, Carlsbad, California, USA). Gels were blotted to nitrocellulose membrane (Amersham Biosciences, Uppsala, Sweden) for 70 min at 30 V, stained with ponceau (Sigma-Aldrich, Hamburg, Germany), blocked in 5% nonfat milk (AppliChem GmBH, Darmstadt, Germany) in 1X TBST (TBS containing 0.1% Tween-20) for 1 h at RT and incubated with respective primary antibodies (listed in Table 2) diluted in 5% BSA for overnight at 4 °C.

**Table 2:** Primary antibodies used for western blot

No	Name	Company and catalog number	Dilution used
1	CD9	Abcam, ab92726	1:500
2	CD63	Abcam, ab68418	1:1000
3	Calreticulin	Cell Signaling Technology, #2891S	1:1000
4	Integrin-b1	Cell Signaling Technology, #9699	1:500
5	TSG101	Abcam, ab83	1:500
6	Ras^G12D^	Cell Signaling Technology, #14429	1:1000

The next day, membranes were washed thrice in 1X TBST, each for 10 min, and were incubated for 1 h at RT with secondary antibodies (Anti-rabbit IgG, HRP-linked Antibody #7074 or Anti-mouse IgG, HRP-linked Antibody #7076, Cell Signaling Technology, Massachusetts, USA, 1:1000 dilution). Membranes were then washed thrice in 1X TBST for 10 min each. Bands were visualized with Immobilon Western Chemiluminescent HRP substrate (Milipore, Darmstadt, Germany) using Fusion FX7 imaging system (Vilber, Marne-la-Vallée cedex 3, France) and analyzed with Image J software (Java-based image processing program developed by National Institutes of Health) for quantification.

### RNA sequencing and analysis

The eluted EV RNAs were first analyzed for their integrity and concentration using Agilent Fragment Analyzer 5200™ with DNF-472 High Sensitivity RNA Analysis Kit, 15 nt (Agilent Technologies, Santa Clara, California, United States). A range of 1 ng–2 μg RNA was used for complementary DNA (cDNA) synthesis as a preparation for EV RNA sequencing (RNA-seq) libraries with SMARTer smRNA-Seq Kit for Illumina (Takara Bio Inc, Mountain View, California, USA) and were sequenced on an Illumina sequencing platform (NextSeq® 500/550 Mid Output Kit v2, San Diego, California, USA) with run configurations of single read, read 1:51 cycles, index 1:8 cycles, index 2:8 cycles and an average of 3.7 million reads per sample.

Raw reads were first converted from bcl to fastq format using bcl2fastq (v2.20.0.4.422) and subsequently filtered using FastQ Screen to remove potential contaminations by microorganisms or artefacts due to technical issues. The reads were mapped to a phase II reference genome of the 1000 Genomes Project. The R software package ‘DESeq2’ using the read counts generated from the RNA-seq was employed to analyze the differential EV gene expression levels between long-term and short-term survivors in the discovery cohort. EV mRNAs with absolute log2 fold-change (Log2FC) > 1 and adjusted* p* value < 0.05 were used to construct a heatmap and further evaluated by receiver operating characteristic (ROC) curves to identify candidate EV mRNAs based on the area under the curve (AUC) for subsequent signature establishment. ROC curves are typically used for diagnostic performance evaluation to discriminate diseased from normal cases based on the AUC with value near to 1 representing a good measure of separability while value near to 0 indicating poor measure of separability [16]. The use of this method in our case helped to identify candidates that were capable of distinguishing between long-term and short-term survivors, since the study first started with the two groups of patients with extremely different OS.

Gene Set Enrichment Analysis (GSEA) was conducted for biological processes analysis of the EV mRNAs between long-term and short-term survivors. For GSEA, 50 hallmark gene sets from Molecular Signatures Database v7.5.1 (MSigDB, https://www.broadinstitute.org/gsea/msigdb/index.jsp) were downloaded and ‘clusterProfiler’ package was used [17]. The default parameters were used to identify significantly enriched gene sets.

### Digital droplet polymerase chain reaction (ddPCR)

To evaluate the potential candidate biomarkers obtained from RNA-seq, multiplex ddPCR was employed to ensure the candidate biomarkers could be validated on a different platform that is more clinically relevant. 1 ng of EV total RNAs were first reversed transcribed into cDNAs using SuperScript™ IV VILO™ master mix (Thermo Scientific, Darmstadt, Germany) according to the manufacturer’s protocol (without ezDNase enzyme treatment). Briefly, the reaction mixtures were incubated at 25 °C for 10 min, 50 °C for 10 min and followed by 85 °C for 5 min. The resulted cDNAs were stored at − 20 °C until further use for the ddPCR experiments. The PrimePCR probe assay for the respective targets (Bio-Rad, Hercules, CA, USA) used in this study is listed in Table 3.

**Table 3:** Targets used for multiplex ddPCR

No	Gene name	Unique assay ID	Fluorophore
1	*AL359195.1*	qHsaCEP0032455	HEX
2	*MAPK8*	qHsaCIP0027490	HEX
3	*MIPOL1*	qHsaCEP0052771	Cy5
4	*MPP4*	qHsaCEP0049739	Cy5
5	*ULK2*	qHsaCIP0030289	FAM
6	*PPP1R12A*	qHsaCIP0027906	HEX
7	*SCN7A*	qHsaCEP0051964	Cy5
8	*SGCD*	qHsaCEP0053985	Cy5
9	*ZBTB8B*	qHsaCEP0055910	HEX
10	*ZNF266*	qHsaCEP0054317	FAM

The ddPCR experiments were performed using 3-color Naica® system for Crystal Digital PCR™(Stilla Technologies, Paris, France) where three keys steps happened on a single chip: (1) partitioning of samples into droplets; (2) thermal cycling; and (3) fluorescence image acquisition of the amplified targets. Briefly, the PCR reaction mixtures comprised 5 μl amplified DNAs, 1.35 μl of 20X PrimePCR probe assay for each target, 5 μl of the 5X Perfecta multiplex qScript toughmix (Quantabio, Beverly, Massachusetts, United States), 2.5 μl of 1 mM fluorescein (VWR, Radnor, Pennsylvania, United States) and top up with nuclease-free water to obtain a final volume of 27 μl. The PCR amplification was carried out with 25 μl of the prepared PCR reaction mixtures loaded to the Sapphire chips (Stilla Technologies, Paris, France) on the Naica Geode based on the following conditions: 95 °C for 10 min (enzyme activation), followed by 60 cycles of 95 °C for 30 s (denaturation) and 58 °C for 90 s (annealing and extension). Series of negative controls were used to determine the limit of blank for each target. The PrimePCR™ template for each target served as positive control. Fluorescence signal quantification was performed by Naica Prism3 acquisition channels (FAM, HEX and Cy5). The analysis of the generated images of the droplet crystals was done with the Crystal Miner software and the output was expressed as the number of mRNA copies per μl of the target molecule in the input reaction (cp/μl).

### Data analysis

All the analyses in this study were performed using R software version 4.1.2 (‘DESeq2’, ‘pROC’, ‘survival’, ‘survminer’ and ‘ggDCA’ packages) and GraphPad Prism 8.0.1. The differentially expressed RNAs from RNA-seq were analyzed using the ‘DESeq2’ package. ‘pROC’ package was used to generate ROC curves in the discovery cohort for candidate EV mRNA selection. ‘ggDCA’ R package was used to perform decision curve analysis (DCA) for evaluating the prognostic value of the signature and the current standard marker.

Kaplan–Meier curves were used extensively in the study to estimate the survival of PDAC patients under different variables of interest. Firstly, to evaluate the prognostic significance of the candidate EV mRNAs on the PDAC patients in the training cohort, the expression levels of each candidate obtained from ddPCR were first assigned into high and low levels based on their optimal cut-off value generated by ‘surv_cutpoint’ function in the ‘survminer’ R package. Subsequently, Kaplan–Meier curves and univariate Cox proportional hazard regression model were used to assess the correlation between the candidate mRNAs and OS of the PDAC patients. Similarly, the same method was used to estimate the correlation between the plasma EV total RNA concentration and OS of the PDAC patients. Thirdly, Kaplan–Meier curves were also employed to assess the correlation between the EV mRNA signature and the OS of the patients from both training and validation cohorts. To construct a prognostic EV mRNA signature, a stepwise Cox proportional hazard regression model was used to investigate the effect of the candidate EV mRNAs from the training cohort on OS of the PDAC patients. The risk score of each patient was calculated using the formula: risk score = expression of mRNA1* coefficient mRNA1 + exp mRNA2* coef mRNA2 + exp mRNA3* coef mRNA3 + ….This is based on the expression of mRNAs and Cox regression coefficients. By comparing the risk score with the optimal cut-off value, the training cohort was divided into a high-risk and a low-risk group. With the two groups of high-risk and low-risk, ‘survival’ R package was used to test if there were prognostic differences between the two groups in the training cohort. The same procedures of calculating risk score and group separation were applied to the multi-center validation cohort and the combined cohort of training and validation cohorts. Univariate Cox proportional hazard regression was also applied additionally to all the generated Kaplan–Meier curves to investigate the effect of the candidate EV mRNAs or signature on survival probability by calculating the hazard ratio (HR) and 95% confidence interval (CI). An HR less than 1 indicates decreased hazard for the survival probability in the high-level group compared with the low-level group, and vice versa.

The independent prognostic effects of the constructed signature and other clinical parameters on patient survival were assessed by univariate and multivariate Cox regression analyses.

For missing data management, there were samples without information of tumour stage (*n* = 2), grade (*n* = 28) and CA19-9 (*n* = 63) that could not be used for further analyses.

### Statistical analysis

Statistical significance for Kaplan–Meier curves was performed by Log-rank test. Statistical significance for the rest of the graphs was calculated by either unpaired two-tailed student’s *t*-test or two-tailed Mann–Whitney *U* test after testing with Shapiro–Wilk test to determine the data normality. *p* < 0.05 was considered statistically significant.

## Results

### Transcriptomic profiling reveals differentially-expressed mRNAs in the plasma EVs between long-term and short-term survivors

**Fig. 1:**
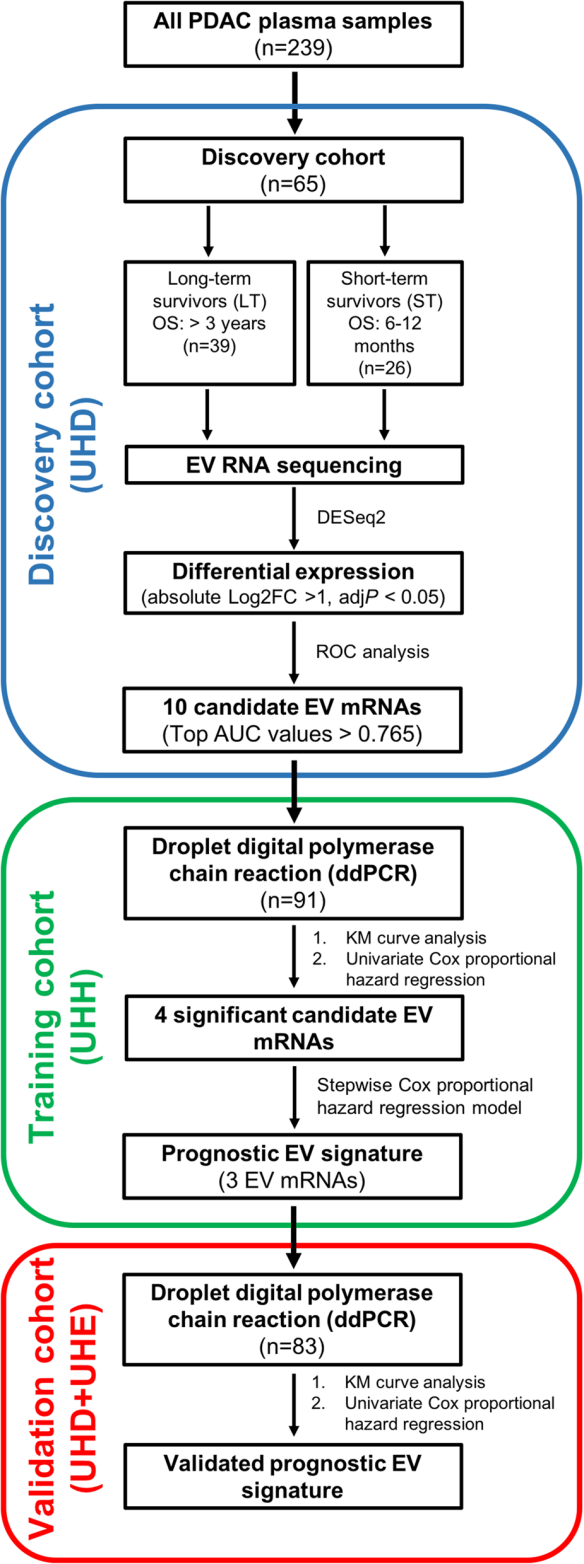
Overview of the study design for the identification of plasma EV mRNA signature as a biomarker for the prognosis of PDAC patients. PDAC: pancreatic ductal adenocarcinoma; OS: overall survival; EV: extracellular vesicles; DESeq2: a R software package for differential gene expression analysis of RNA sequencing data; absolute Log2FC: absolute Log2 fold-change; ad*jP*: adjusted* p* value; ROC: receiver operating characteristic curve; AUC: Area under the curve; KM: Kaplan–Meier; UHD: University Hospital Dresden; UHH: University Hospital Heidelberg; UHE: University Hospital Erlangen

**Fig. 2:**
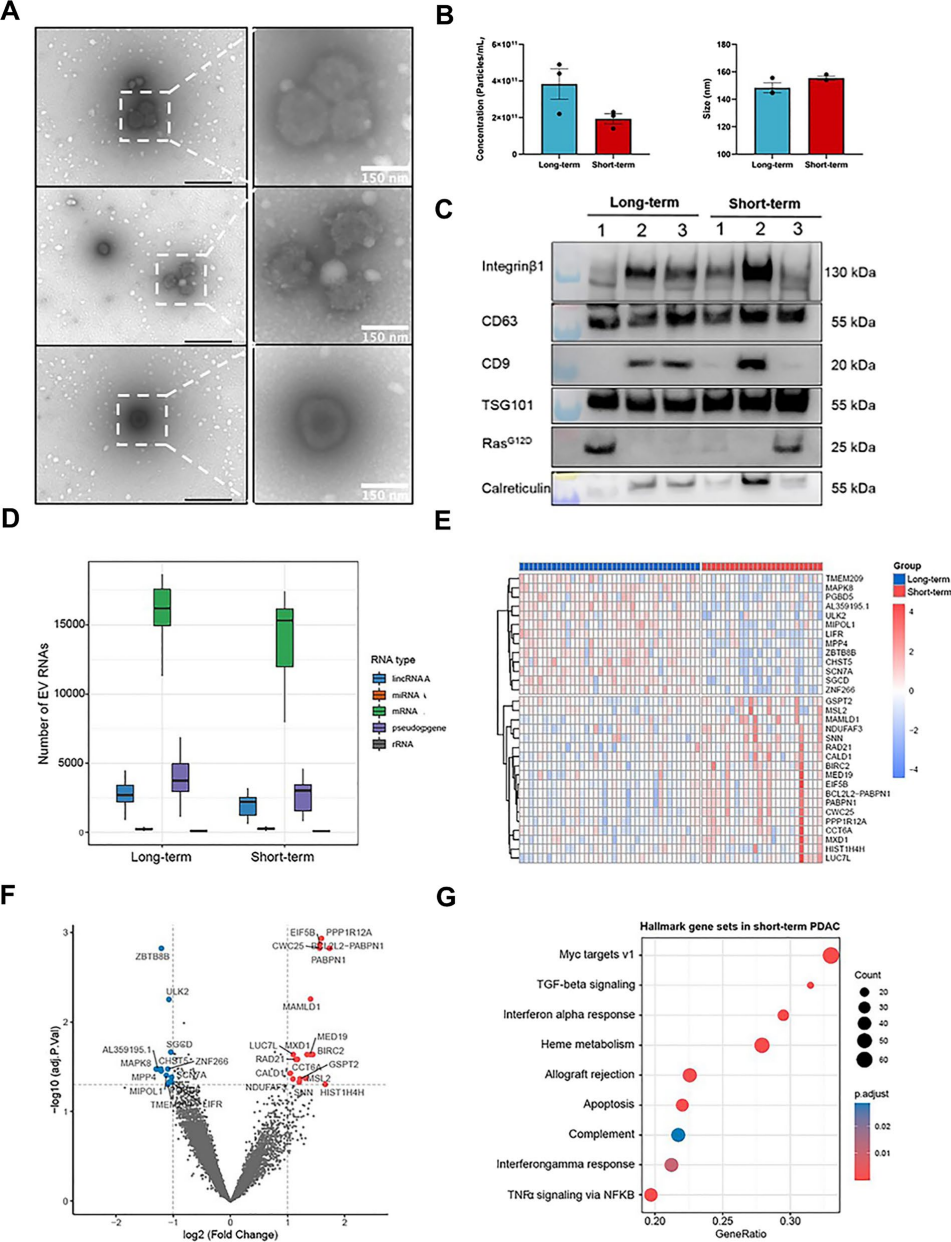
The expressional landscape of plasma EV RNAs in long-term and short-term PDAC survivors from the discovery cohort. **A** Negative-stain transmission electron microscopy (TEM) of extracellular vesicles (EVs) isolated from PDAC patients. Representative figures showed the heterogenity of EVs from plasma of PDAC patients. **B** Nanoparticle tracking analysis (NTA) for the concentration and size of the EVs from long-term and short-term PDAC survivors. **C** Western blot analysis of EV- and non-EV protein levels in the EVs isolated from PDAC patients. **D** Distribution of all EV RNAs based on the results from RNA sequencing of long-term and short-term PDAC patients. LincRNA: Long intervening noncoding RNA; mRNA: messenger RNA; miRNA: micro RNAs; rRNA: ribosomal RNA; pseudogene: non-functional segments of DNA. **E** Heatmap of differentially expressed EV mRNAs between long-term and short-term survivors. Each row represents an EV mRNA whereas each column denotes a patient sample. **F** Volcano plot displayed differentially expressed genes that were significantly upregulated (red dots) or down-regulated (blue dots) when compared with short-term survivors (adjusted *p* value < 0.05) and absolute Log2 fold-change > 1). **G** Gene Set Enrichment Analysis (GSEA) showed the differentially expressed EV mRNAs from the short-term survivors significantly enriched in the Hallmark pathways compared with the long-term survivors (adjusted *p* value < 0.05). MYC: Master regulator of cell cycle entry and proliferative metabolism; TGF Beta: Transforming growth factor beta 1; TNFα: Tumour necrosis factor alpha; NFκB: Nuclear factor kappa B

As a basis for further characterization and identification of plasma EV biomarkers, a comprehensive EV isolation method comparison was performed to select the best EV isolation method for EV RNA biomarker discovery. The results showed that column-based membrane affinity (exoRNeasy Serum/Plasma Midi kit) was found to be the best EV isolation method after considering the EV recovery, purity and RNA concentration (Additional file 1: Fig. S2, Additional file 1: Table S1). With the column-based membrane affinity method to isolate circulating EVs from plasma, the study proceeded as illustrated in Fig. 1 and Table 1. As a representation, EVs from long-term and short-term survivors in the discovery cohort were subjected to characterization by TEM, NTA and western blot according to the MISEV 2018 guidelines [18]. The results of TEM analysis displayed a heterogenous population of particles exhibiting an artificial cup-shaped morphology due to the fixation during the handling process (Fig. 2A). Secondly, NTA showed that the mean diameters distribution of particles was found to be around 150 nm (148.4 ± 3.62 nm for long-term survivors; 155.4 ± 1.32 nm for short-term survivors) and the concentration of the particles was an average of 3.83 × 10^11^ ± 8.29 × 10^10^ particle/ml for long-term survivors and 1.93 × 10^11^ ± 2.73 × 10^10^ particle/ml for short-term survivors, which was not statistically significant between the two groups (Fig. 2B). Additionally, EVs were subjected to protein composition characterization by western blot. For transmembrane proteins, Integrin b1 and CD63 were detected in the fractions from all the patients, regardless of long-term or short-term survivors (Fig. 2C). However, not all the eluted fractions from the tested patients contained CD9 protein markers, implying EV inter-individuality difference among different PDAC patients. As for the category of cytosolic proteins related to multivesicular bodies (MVBs), TSG101 was observed in the fractions of all the patients from the two groups. It is noteworthy that KRAS^G12D^ protein was clearly evident in the fractions of one patient from each group, suggesting that the isolated EVs specific to PDAC tumour cells, as > 80% of PDAC patients contain mutated KRAS and of which 28% patients carry *KRAS* point mutation (G>A) [19]. This indicates the fact that mutated proteins from cancer cells can be packaged into EVs and present in the circulation. On the other hand, calreticulin, a marker of ER, could still be detected in some of the patient fractions with varying degree, which suggest the possibility of the presence of other non-vesicular components [20] in the eluted EV fractions. Taken together, the morphology of cup-shaped, size of around 150 nm and the presence of EV markers from the categories of transmembrane and cytosolic proteins suggest the identity of the isolated EVs.

To identify plasma-derived EV mRNAs that could be used as prognostic biomarkers for PDAC patients, the EV total RNAs isolated from the PDAC patients in the discovery cohort (long-term, *n* = 39 and short-term survivors, *n* = 26) were subjected to RNA-seq for transcriptomic profiling. Distinct types of RNAs such as long intergenic non-coding RNAs (lincRNAs), microRNAs (miRNAs), messenger RNAs (mRNAs), pseudogenes and ribosomal RNAs (rRNAs) were detected in the plasma EVs of all patients (Fig. 2D). Among the different types of RNAs, approximately 15,696 detected genes belong to mRNAs (Fig. 2D). Based on the RNA-seq count data, 31 differentially expressed mRNAs between long-term and short-term PDAC survivors were identified with criteria of absolute Log2FC > 1 and adjusted *p* value < 0.05, as illustrated in the hierarchical clustering heatmap (Fig. 2E, Additional file 1: Table S2). The overall pattern of the mRNA expression was distinctly different between the long-term and short-term PDAC survivors (Fig. 2E). To clearly display the expression levels of the differentially expressed mRNAs, a volcano plot was shown to indicate upregulated (red dots) or down-regulated mRNAs (blue dots) in the short-term survivors compared with long-term PDAC survivors (Fig. 2F). It could be observed that protein phosphatase 1 regulatory subunit 12A (*PPP1R12A*), eukaryotic translation initiation factor 5B (*EIF5B*), Spliceosome-Associated Protein Homolog (*CWC25*) were statistically highly upregulated whereas zinc finger and BTB domain containing 8B (*ZBTB8B*) and Unc-51 like autophagy activating kinase 2 (*ULK2*) were significantly down-regulated in the short-term survivors. In addition, to gain insights into the biological cellular processes of these differentially expressed mRNAs, GSEA was performed. The GSEA revealed these plasma EV-packaged mRNAs from short-term survivors were significantly enriched in several key hallmark cancer-related pathways, i.e., Myc, TGF-b, interferon-a, interferon-g, TNFα via NFkB signalling pathways, suggesting that these EV mRNA cargoes were related to specific cellular processes and reflect tumour biology (Fig. 2G).

### Analysis of top-ranked mRNAs establishes circulating EV mRNA signature for PDAC prognosis

**Fig. 3:**
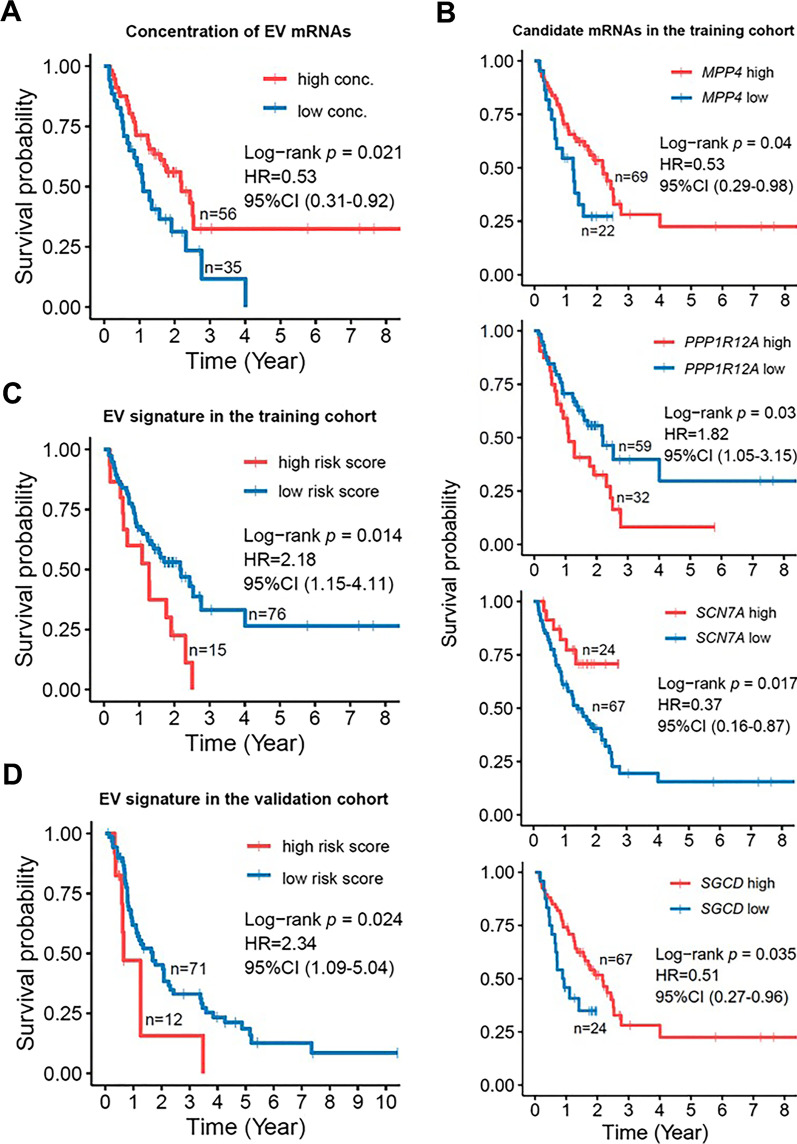
Prognostic EV mRNA signature establishment and validation in PDAC patients. **A** The prognostic correlation of plasma EV total RNA concentration from patients in the training cohort were assessed by Kaplan–Meier survival analysis. **B** Kaplan–Meier survival curves for the candidate EV mRNA expression levels of the patients in the training cohort. The expression data were obtained from the digital droplet polymerase chain reaction (ddPCR) analysis. *MPP4*: Membrane Palmitoylated Protein 4; *PPP1R12A*: Protein Phosphatase 1 Regulatory Subunit 12A; *SCN7A*: Sodium Voltage-Gated Channel Alpha Subunit 7; *SGCD*: Sarcoglycan Delta. **C-D** The prognostic performance of EV mRNA signature consisted of *PPP1R12A*, *SCN7A*, and *SGCD* in the training and validation cohort were assessed by Kaplan–Meier survival analysis. Hazard ratio (HR) and 95% confidence interval (CI) were generated from univariate Cox proportional hazard regression analysis. Statistical differences were analyzed with log-rank test. *p* < 0.05 is considered statistically significant

To select the candidate mRNAs for EV signature establishment, ROC analysis was used to choose the top ranked mRNAs based on the AUC values, as this could give us the most distinct candidates between the two groups of long-term and short-term survivors. As a result, 10 candidate mRNAs were selected, i.e., *ZBTB8B*, sodium voltage-gated channel alpha subunit 7 (*SCN7A*), zinc finger protein 266 (*ZNF266*), *AL359195.1*, *ULK2*, membrane palmitoylated protein 4 (*MPP4*), sarcoglycan delta (*SGCD*), mitogen-activated protein kinase 8 (*MAPK8*), mirror-image polydactyly 1 (*MIPOL1*), *PPP1R12A* (Additional file 1: Fig. S3) and trained in a training cohort (*n* = 91 PDAC patients) by ddPCR (Fig. 1). For performing ddPCR, the concentration of the EV total RNAs from all the patients was measured in order to use the same concentration for cDNA synthesis. It was of interest to examine if the concentration of the plasma EV total RNAs was correlated with the OS of the PDAC patients in the training cohort. Hence, the EV concentration of the PDAC patients was divided into two groups of high and low levels using the optimal cut-off calculated by the R package. Interestingly, the concentration of plasma EV total RNAs was positively correlated to the OS by Kaplan–Meier analysis (log-rank *p* = 0.021) and univariate Cox proportional hazard regression analysis (*p* = 0.023, HR 0.53, 95% CI 0.31–0.92, Fig. 3A). The ddPCR results of the training cohort revealed 4 candidate mRNAs, i.e., *MPP4*, *PPP1R12A*, *SCN7A* and *SGCD* exhibited significant correlations with OS of the PDAC patients (Fig. 3B). In particular, patients with higher expression levels of *MPP4* (log-rank *p* = 0.04), *SCN7A* (log-rank *p* = 0.017) and *SGCD* (log-rank *p* = 0.035) displayed a superior OS, whereas those with higher expression levels of *PPP1R12A* (log-rank* p* = 0.03) experienced rather an inferior OS. Meanwhile, univariate Cox proportional hazard regression analyses revealed that *MPP4* (*p* = 0.044, HR 0.53, 95% CI 0.29–0.98), *SCN7A* (*p* = 0.022, HR 0.37, 95% CI 0.16–0.87) and *SGCD* (*p* = 0.038, HR 0.51, 95% CI 0.27–0.96) were favourable predictors of OS, while *PPP1R12A* (*p* = 0.032, HR 1.82, 95% CI 1.05–3.15) was an unfavourable predictor of OS (Fig. 3B). With the intention of constructing an EV signature, a stepwise Cox proportional hazard regression model was used to evaluate the possibility of *MPP4*, *PPP1R12A*, *SCN7A* and *SGCD* to be used as a signature. The results showed that 3 out of 4 candidate mRNAs, i.e., *SCN7A*, *SGCD* and *PPP1R12A* were successfully selected by the stepwise Cox proportional hazard regression model to form a prognostic signature for predicting the survival of PDAC patients in the training cohort. The hazard regression model involved risk scores that were calculated by the following formula: risk score = *SCN7A* expression * (− 0.7860) + *SGCD* expression * (− 0.7247) + *PPP1R12A* expression * (0.6447). As a result, the patients in the training cohort with risk scores scaled between − 4.4998 and + 7.8552 were divided into the high-risk group (*n* = 15) and low-risk group (*n* = 76) based on the optimal cut-off value. It could be noticed that the patients with high-risk scores were significantly correlated with inferior OS as demonstrated by Kaplan–Meier analysis (log-rank *p* = 0.014) and univariate Cox proportional hazard regression analysis (*p* = 0.017, HR 2.18, 95% CI 1.15–4.11) (Fig. 3C), suggesting the potential prognostic application of this novel EV mRNA signature.

To confirm its prognostic implication, the EV 3-mRNA signature was further evaluated on an independent multi-center validation cohort (*n* = 83). The risk scores of these PDAC patients were calculated using the same formula in the training cohort and were scaled between − 0.5611 and + 7.8583. Similarly, the patients were assigned to the high-risk group (*n* = 12) and low-risk group (*n* = 71) based on the optimal cut-off value. Importantly, the signature was confirmed to be a strong predictor for the OS of PDAC patients in an independent cohort by Kaplan–Meier analysis (log-rank *p* = 0.024) and univariate Cox proportional hazard regression analysis (*p* = 0.029, HR 2.34, 95% CI 1.09–5.04) (Fig. 3D). Collectively, these findings suggest that the EV 3-mRNA signature could be used for risk stratification to predict the prognosis of PDAC patients.

### The EV 3-mRNA signature has improved prognostic performance in combination with tumour stage

**Fig. 4:**
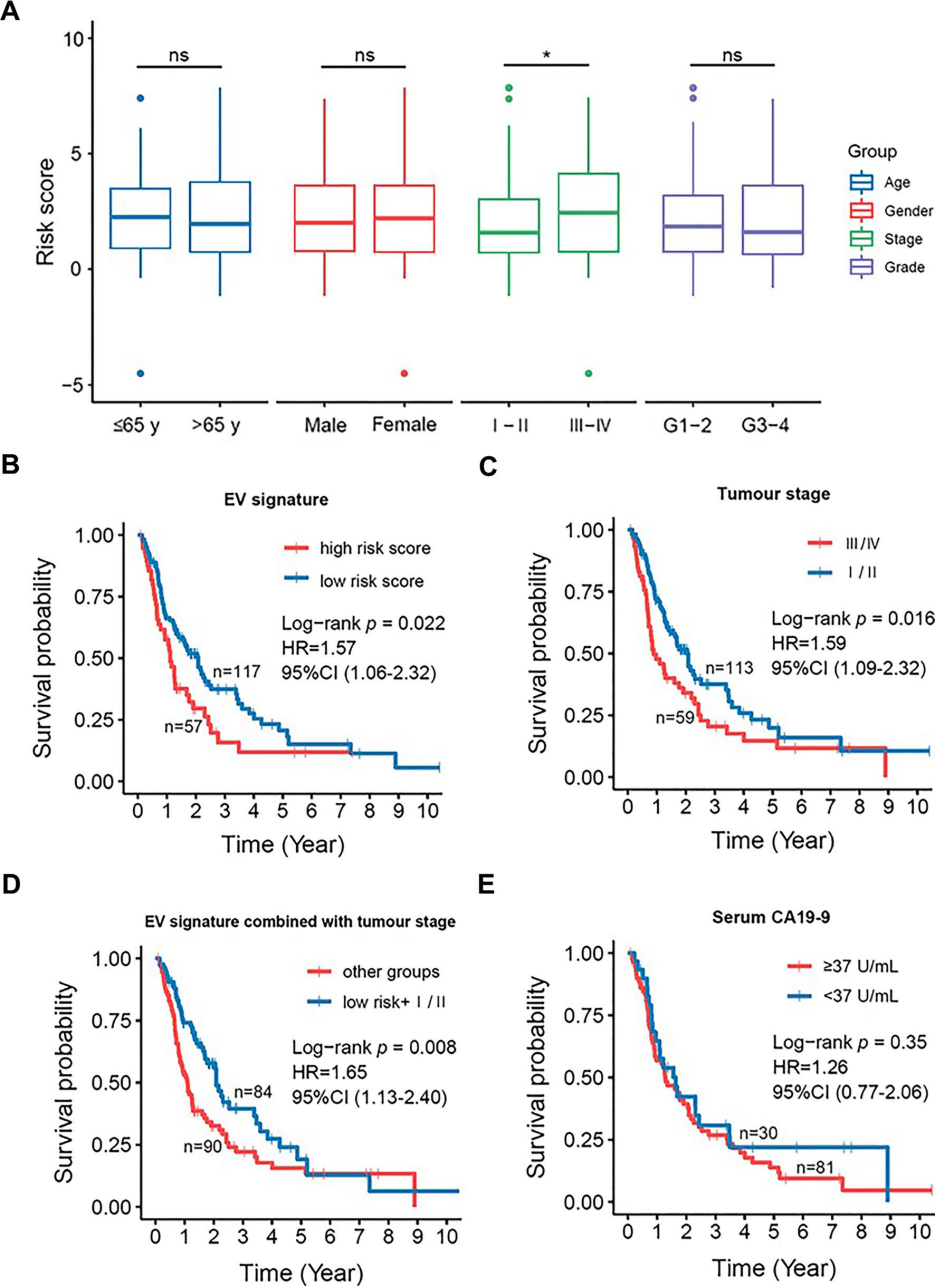
The EV 3-mRNA signature is an independent prognostic biomarker for overall survival (OS) prediction of PDAC patients. **A** Box plots analysis showed the distribution of signature-associated risk score in PDAC patients from both training and validation cohorts categorized into subgroups of age (≤ 65 *y* = 46; > 65 = 75), gender (male = 60; female = 61), tumour stage (stage I–II = 113; stage III–IV = 59) and grading (G1–2 = 77; G3-4 = 69). Statistical differences were analyzed by Mann–Whitney *U* test after Shapiro–Wilk test. *p* < 0.05 is considered statistically significant. ns = not significant. **B–D** Kaplan–Meier analysis of OS of PDAC patients from both training and validation cohorts based on the risk scores of EV mRNA signature, tumour stage and combined EV mRNA signature and tumour stage. Hazard ratio (HR) and 95% confidence interval (CI) were generated from univariate Cox proportional hazard regression analysis. Statistical differences were analyzed with log-rank test. *p* < 0.05 is considered statistically significant. **E** The prognostic performance of serum CA19-9 in the combined training and validation cohorts of PDAC patients. The commonly routine used cut-off value of 37 units per milliliter (U/ml) was employed in this study. CA19-9: Carbohydrate antigen 19-9. Hazard ratio (HR) and 95% confidence interval (CI) were generated from univariate Cox proportional hazard regression analysis. Statistical differences were analyzed with log-rank test. *p* < 0.05 is considered statistically significant

To further evaluate its prognostic ability and clinical utility, the EV 3-mRNA signature was examined for its relationship with common clinical features in a combined cohort consisting of training and validation cohorts. The results showed that the signature was not correlated with age (*n* for ≤ 65 years old = 67; *n* for > 65 years old = 107), gender (*n* for male = 87; *n* for female = 87), and cancer grade (*n* for G1–G2 = 77; *n* for G3–G4 = 69) of the PDAC patients (Fig. 4A). Interestingly, the risk score of the signature was found to be significantly higher in stage III–IV patients compared with patients in stage I–II (*n* for stage I–II = 113; *n* for stage III–IV = 59), indicating that the signature correlates with tumour progression (Fig. 4A). Furthermore, analysis of Kaplan–Meier curves demonstrated that our EV 3-mRNA signature and tumour stage independently displayed significant correlations with OS of the PDAC patients in the combined cohort with log-rank *p* = 0.022 and log-rank *p* = 0.016, respectively (Fig. 4B and C). However, age, gender, grade, neoadjuvant and adjuvant therapies did not show significant correlations with the OS of the patients in the combined cohort (Additional file 1: Fig. S4). Although CA19-9 is also commonly used for prognostic application in addition to diagnosis [21, 22], it should be noted that CA19-9 had no significant prognostic ability in our combined cohort (Fig. 4E). Furthermore, DCA demonstrated that the EV 3-mRNA signature displayed stronger prognostic benefit than CA19-9 for predicting 1-year, 2-year and 3-year survival of PDAC patients (Additional file 1: Fig. S5). However, the combination of EV 3-mRNA signature-based risk and CA19-9 showed higher prognostic value when the threshold probability was higher than 0.723 whereas the prognostic value of the EV mRNA signature remained better than the CA19-9 or the combination of both when the threshold probability was between 0.685 and 0.723, suggesting that the EV mRNA signature has better prognostic value either individually or in combination with CA19-9 (Additional file 1: Fig. S5). Next, it was of high interest to investigate if our EV signature could surpass or complement to tumour stage, which is one of the commonly used prognostic factors in the clinical scene. The signature and tumour stage were thus subjected to further Kaplan–Meier analysis in a single and/or combination manner. Interestingly, the combination of EV signature and tumour stage represented a higher prognostic value (log-rank *p* = 0.008) than the signature (*p* = 0.022) or tumour stage (*p* = 0.016) alone (Fig. 4D). In addition, univariate Cox proportional hazard regression analysis showed *p* value of 0.009, HR  1.65, 95% CI  1.13–2.40 for the combined EV signature and tumour stage (Fig. 4D), suggesting that the EV signature could complement to the current prognosis of PDAC patients.

To evaluate if the EV 3-mRNA signature is an independent predictor of OS, univariate and multivariate Cox regression analyses were performed. Based on the results in Table 4, the EV signature was indeed found to be an independent prognostic factor (*p* = 0.035) after considering age, gender, tumour stage, cancer grade, resection margin, neoadjuvant chemotherapy and adjuvant chemotherapy (Table 4). Taken together, these data indicated that the EV 3-mRNA signature was a robust and independent predictor of the prognosis of PDAC patients. In addition, the EV signature can complement the tumour stage to yield superior prognostic performance for the prediction of the survival of PDAC patients.

**Table 4:** Univariate and multivariate Cox regression analysis of the EV 3-mRNA prognostic signature in the combined training and validation cohort

Variables		Univariate Cox			Multivariate Cox		
		HR	95% CI	*p* value	HR	95% CI	*p* value
Age							
≤ 65y/ > 65y	*N* = 174	0.78	0.54–1.14	0.204	–	–	–
Gender							
Female/Male	*N* = 174	0.79	0.55–1.15	0.226	–	–	–
Stage							
I-II/III-IV	*N* = 172	1.59	1.09–2.32	0.017	1.25	0.81–1.92	0.320
Grade							
G1–2/G3–4	*N* = 146	1.22	0.81–1.85	0.346	–	–	–
Resections							
R0/R1 R0/R2	*N* = 168	1.11 3.23	0.74–1.67 1.67–6.24	0.618 < 0.001	1.08 3.11	0.71–1.63 1.41–6.87	0.735 0.005
Neoadjuvant chemotherapy							
No/yes	*N* = 107	0.78	0.43–1.41	0.407	–	–	–
Adjuvant chemotherapy							
No/yes	*N* = 113	0.76	0.37–1.56	0.458	–	–	–
Risk score							
Low/high	*N* = 174	1.57	1.06–2.32	0.024	1.57	1.03–2.39	0.035

## Discussion

Liquid biopsies show great promises for early cancer detection, monitoring tumour progression and treatment responses since the last decade, with the help of the continued evolving advanced technologies. This is of utmost importance for PDAC due to the secondary retroperitoneal location of the pancreas and morbidity associated with tissue sampling. Among the different types of liquid biopsies, billions of EVs present in the systemic circulation carry important cargoes that provide adjunctive or potentially superior diagnostic, prognostic or predictive biomarkers. Despite previous EV RNA biomarker studies [12, 13, 23], the EV RNA research landscape showing the potential of EV mRNAs for PDAC prognosis remains elusive, especially for survival prediction.

In this study, the aim was to identify non-invasive EV mRNA biomarkers for survival prediction of PDAC patients after surgery. Since plasma remains the most accessible source for liquid biopsies and is depleted of platelet-derived EVs, it was hypothesized that plasma EVs contained mRNA cargoes that could be employed as prognostic biomarkers for PDAC patient survival prediction. Here, we performed a large-scale, comprehensive analysis of circulating plasma EV transcriptomes from a total of 239 PDAC patients in 3 different cohorts (discovery, training and validation) to identify prognostic EV mRNAs for risk stratification and survival prediction of PDAC patients. Before delving into the research topic of interest directly, this study first performed a comparison of the different EV isolation methods to gain an idea of the best method for our EV mRNA biomarker study. It was shown that column-based membrane affinity yielded the highest EV RNA concentration and ratio of particle/protein concentration compared with precipitation and ultracentrifugation. These results are in accordance to the current findings that precipitation tends to precipitate many other non-vesicular proteins that could result in high protein concentration and low purity [24, 25]. Although differential ultracentrifugation has been commonly used by many EV researchers in the field, it is widely known that the recovery of EVs from ultracentrifugation is generally low [26, 27]. Membrane affinity isolation method allowed for short processing time and high RNA concentration from a low input volume (0.5–1 ml). Hence, membrane affinity was selected as the best EV isolation method for this biomarker discovery study.

A novel EV 3-mRNA prognostic signature for predicting the survival of PDAC patients was identified and validated successfully in this study. With a discovery cohort consisting of PDAC patients with both long-term and short-term survival, transcriptomic profiling identified differentially expressed genes that allowed for screening of top-ranked candidates to be selected for testing on a training cohort. To confirm the identity of each mRNA candidate and for reproducibility purpose, the commercially available target probes specific to several predicted target binding sites were used for ddPCR validation. It was observed that 4 candidate mRNAs were successfully trained on the training cohort. The EV signature was established based on 3 out of the 4 mRNAs and showed significant prognostic performance for predicting the OS of PDAC patients in the training and validation cohorts. Moreover, in conjunction with tumour stage, the EV 3-mRNA signature was demonstrated to provide a superior risk stratification for PDAC patients than the EV signature or tumour stage alone, implying synergistic potential of the EV signature and tumour stage, a typical clinically used prognostic factor. Since cancer is the result of the accumulation of numerous mutations and PDAC is notoriously heterogenous at multiple levels, a particularly pertinent approach would be to collaborate our EV 3-mRNA signature with tumour stage and imaging to predict the survival of the patients from different aspects. Although larger validation cohorts are required to confirm the prognostic effect of the EV 3-mRNA signature and to explore its predictive value, the current study has evidently demonstrated the use of EV mRNAs from circulating plasma as liquid biopsy that may support clinical implementation.

Plasma EV mRNAs from cancer patients reflect the systemic effects of cancer cells and the cancer-associated changes resulting from the developing primary tumour, its surrounding tumour microenvironment, distant organs and the immune system. The prognostic EV signature identified in this study comprised of 3 mRNAs: *PPP1R12A*, *SCN7A* and *SGCD*. PPP1R12A, also known as myosin phosphatase-targeting subunit 1 (MYPT1), is essentially a phosphatase that inactivates myosin by dephosphorylating regulatory light chain (RLC) [28]. Proper expression levels of PPP1R12A are critical for RLC phosphorylation and actin assembly in order to maintain normal cellular functions of cell division and cell migration [29]. The fact that its presence is almost ubiquitous, i.e., cytoplasm, cytoskeleton, nucleus, mitochondria and plasma membrane, the functions of PPP1R12A are believed to be not just a myosin phosphatase [30]. For instance, it has been found to be a downstream target of RhoA/ROCK signalling mediating many different cellular processes [31]. In the context of pancreatic cancer, it has been shown that the expression of PPP1R12A was significantly higher in the tumour tissues compared with the normal tissues at both the mRNA and protein levels [32]. On top of that, the expression of PPP1R12A was also detected in cancer that was invading pancreatic nerves and regional lymph nodes [32], indicating that PPP1R12A may play a role in the metastasis of pancreatic cancer. Besides, it was reported that a significantly higher cytoplasmic and membranous PPP1R12A expression was found in pancreatic compared to biliary, colon breast and lung cancers [32], suggesting the specificity of PPP1R12A to pancreatic cancer over other solid cancers. Secondly, SCN7A, also known as SCN6A, encodes a voltage-dependent sodium channel of the excitable membrane in cell types such as neurons and muscles for membrane depolarization [33]. Recently, single-nucleus RNA sequencing of human pancreas tissues identified a cluster of Schwann cells expressing *SCN7A*, implying possible functions in axonogenesis, synapse assembly and organization [34]. Moreover, an in silico analysis of TCGA pancreatic cancer dataset reported high expression levels of *SCN7A* in the high immune and stromal score groups, which were associated with better OS of the patients [35], suggesting the prognostic value of *SCN7A* for patients with pancreatic cancer. Although voltage-gated sodium channels have been reported to be involved in metastasis of various cancers [36], the direct role of SCN7A in PDAC tumour cells has not been described thus far. Since the currently available studies demonstrating the presence and role of *SCN7A* in Schwann cells, immune and stromal cells surrounding pancreatic cancer, it was speculated that the role of SCN7A may lie in the tumour microenvironment rather than the tumour cells. As for SGCD, it is a transmembrane protein that connects the muscle fibre cytoskeleton to its surrounding extracellular matrix, preventing damage to the muscle fibre sarcolemma through shearing forces [37]. Similar to SCN7A, the role of SGCD in PDAC has not been elucidated. However, studies have reported its key functions in fibroblasts as a focal adhesion point or profibrotic gene [38, 39]. Taken together, it is conceivable that the EV PPP1R12A may reflect the effect of PDAC cells whereas SCN7A and SGCD may represent essential components from the tumour microenvironment, i.e., immune cells, fibroblasts and Schwann cells. Thus, this EV signature may encompass key players that truly reflect the systemic effect of tumour and its associated microenvironment. Substantial work is still required to understand the origin/source of the EV cargoes, but the current study has provided a promising base for further exploring the specific mRNA cargoes that make the EVs so valuable to liquid biopsies.

While it is obvious that improved diagnostic biomarkers for early diagnosis of PDAC may impact patient survival significantly, no biomarker is ready for clinical implementation after decade of intense investigation and CA19-9 remains the standard routine biomarker for PDAC despite having a sensitivity of 79–81% and specificity of 82–90% [40]. It is hoped that identification of prognostic biomarkers for PDAC patients could open a door towards improved quality of life and overall survival, due to risk-adaptive strategies for optimal therapeutic decision-making. Studies focusing on EV prognostic biomarkers for PDAC emerged only around 5 years ago. For instance, > 5% KRAS mutant allele frequency in circulating exosomes (a subtype of EVs) in patients with metastatic PDAC have been demonstrated to be associated with reduced progression-free survival and overall survival rates, suggesting the potential use of exosomal DNA as liquid biopsy for PDAC patients [8, 41]. Since miRNAs are shown to be relatively more stable in the circulation, several studies have reported different miRNAs as prognostic biomarkers for PDAC such as plasma exosomal miR-451a, portal vein exosomal miR-4525, miR-451a and miR-21 as well as serum exosomal miR-200b [14, 42, 43]. Apart from that, high expression levels of exosomal protein c-Met and PD-L1 from serum samples of PDAC patients were associated with shorter overall survival of resected PDAC patients, indicating that they are negative prognostic factors for PDAC patients [44]. These abovementioned studies show that the investigation of EV prognostic biomarkers is still in the early stage, as the sample size for all the studies above was relatively small (< 100 patients) and the identified markers remain to be further validated for their accuracy and reliability. Our study showing a prognostic EV mRNA signature is currently one of the largest-scale study (*n* = 258) focusing on the identification of prognostic biomarkers for PDAC patients. The study was executed in discovery, training and validation cohorts with the techniques of RNA sequencing and ddPCR, which is a more clinically relevant platform. This has put our study at the forefront of the research field with the potential use towards clinical implementation. Plasma-based EV signature offers the advantage of being non-invasive, allowing for easy serial sampling for monitoring purpose. In addition, EVs provide stability to RNAs in circulation due to their lipid bilayer membrane. For its successful clinical translation, some challenges remain to be overcome. While EV research has progressed exponentially, there is no consensus on the standard EV isolation and detection method for a particular downstream application at the moment. As a result, findings from different studies have shown method- and platform-dependent, rendering generalization of the findings, systematic and meta-analysis difficult. Given that EVs are found to be rather heterogenous [45], it adds additional complexity to apply EV-based biomarkers or signatures to the clinic when EV isolation and detection methods are not standardized, since standardization is paramount for clinical practice. Furthermore, another challenge associated with application of EV-based biomarkers as liquid biopsy is the development of an isolation method with high efficiency and purity. Isolation methods such as column-based membrane affinity, magnetic bead-based immunoaffinity and microfluidic chips have been shown to possess relatively higher efficiency and purity than the commonly used ultracentrifugation [46, 47]. However, more advanced techniques might be more applicable for the routine clinical use. This particular research field will no doubt grow and change as more data arise in the future for successful clinical implementation.

Despite the successful establishment and validation of this EV 3-mRNA signature for PDAC prognosis, it is unavoidable that this study associates with a few limitations. Since the patients in this study were subjected to surgery, the prognostic value of this EV mRNA signature might be applicable only to those PDAC patients at the potentially resectable stage at the time of diagnosis. To have a broader overview of the prognostic value of the EV mRNA signature, it remains to be determined if the signature also accounts for unresectable PDAC patients at the time of diagnosis in a follow-up study. A longitudinal study showing the distribution of the EV 3-mRNA signature over time could provide more insights into the individual and overall trajectory of the signature which may aid in accurate prognoses. According to the latest epidemiological statistics, the incidence rate of pancreatic cancer was highest in Northern America (age-standardized rate (ASR): 8.0 per 100,000 person) followed by Europe (ASR: 7.8 per 100,000 person), which was two-fold and nearly four-fold higher than in Asia (ASR: 4.0 per 100,000 person) and Africa (ASR: 2.3 per 100,000 person), respectively [3], suggesting the wide geographical variation of pancreatic cancer incidence and the underlying associated risk factors. It should be noted that this study consisted exclusively of European patients and thus, generalized application of this EV mRNA signature for other ethnicity groups might be difficult. A follow-up study involving collaboration with hospitals in other continents is necessary to validate the potential use of this EV 3-mRNA signature in other ethnicity groups such as Asians and Africans. Apart from that, it is widely accepted that reprogrammed metabolism by rewired glucose, amino acid and lipid may play a critical role in the progression, treatment and prognosis of PDAC [48]. It is therefore important to consider the relevance of obesity, smoking and alcohol consumption habits, as well as diabetes condition in this EV mRNA signature establishment. However, the incomplete information about body mass index (BMI), smoking, alcohol consumption and diabetes status of our patients in this study render further analysis impossible. Further study focusing on the aspect of PDAC metabolism could be considered to evaluate the correlation between the EV mRNA signature and obesity, smoking, alcohol consumption or diabetes, giving additional perspective of the prognostic value of the EV 3-mRNA signature. Last but not least, further studies on large patient cohorts will be required to confirm the prognostic performance of the EV mRNA signature in order to be applied in the clinic as a standard and routine prognostic test for PDAC patients.

## Conclusions

A large-scale and comprehensive analysis of circulating plasma EV transcriptomes from a total of 239 PDAC patients in 3 different cohorts (discovery, training and validation) enabled us to identify a novel and non-invasive prognostic EV 3-mRNA signature that could be used for risk stratification for survival prediction of PDAC patients. This EV mRNA signature is an independent unfavourable predictor that complements to tumour stage for PDAC prognosis. Our findings fill up the current research gap in liquid biopsy by proofing the feasibility to clinically use a plasma EV mRNA signature for PDAC prognosis.

## Supplementary Information

**Additional file 1**. Supplementary figures (Fig. S1–S5) and tables (Table S1–S2).

## Data Availability

All data generated from this study, if not included in this article, are available from the corresponding authors on reasonable request.

## Abbreviations

PDAC: Pancreatic ductal adenocarcinoma
OS: Overall survival
EV: Extracellular vesicles
ddPCR: Digital droplet polymerase chain reaction
DESeq2: A R software package for differential gene expression analysis of RNA sequencing data
Absolute Log2FC: Absolute Log2 fold-change
ad*jP*: Adjusted* p* value
ROC: Receiver operating characteristic curve
AUC: Area under the curve
KM: Kaplan–Meier
UHD: University Hospital Dresden
UHH: University Hospital Heidelberg
UHE: University Hospital Erlangen
TEM: Transmission electron microscopy
NTA: Nanoparticle tracking analysis
LincRNA: Long intervening noncoding RNA
mRNA: Messenger RNA
miRNA: Micro RNAs
rRNA: Ribosomal RNA
Pseudogene: Non-functional segments of DNA
*EIF5B*: Eukaryotic translation initiation factor 5B
*CWC25*: Spliceosome-Associated Protein Homolog
*ZBTB8B*: Zinc finger and BTB domain containing 8B
*ULK2*: Unc-51 like autophagy activating kinase 2
MYC: Master regulator of cell cycle entry and proliferative metabolism
TGF Beta: Transforming growth factor beta 1
TNFα: Tumour necrosis factor alpha
NFκ B: Nuclear factor kappa B
*MPP4*: Membrane Palmitoylated Protein 4
*PPP1R12A*: Protein Phosphatase 1 Regulatory Subunit 12A
*SCN7A*: Sodium Voltage-Gated Channel Alpha Subunit 7
*SGCD*: Sarcoglycan Delta
HR: Hazard ratio
95% CI: 95% Confidence interval
ZNF266: Zinc finger protein 266
*AL359195.1*: Uncharacterized gene
*MAPK8*: Mitogen-activated protein kinase 8
*MIPOL1*: Mirror-image polydactyly 1
ASR: Age-standardized rate
DCA: Decision curve analysis
