# Supplementary material for: Plasma extracellular vesicle messenger RNA profiling identifies prognostic EV signature for non-invasive risk stratification for survival prediction of patients with pancreatic ductal adenocarcinoma
Source: J Hematol Oncol. 2023 Feb 3;16:7. doi: 10.1186/s13045-023-01404-w (PMC9896775; doi:10.1186/s13045-023-01404-w)
Supplement: Supplementary file 1 — Additional file 1. Supplementary figures (Fig. S1–S5) and tables (Table S1–S2). [file 13045_2023_1404_MOESM1_ESM.docx]

**Supplementary figures**


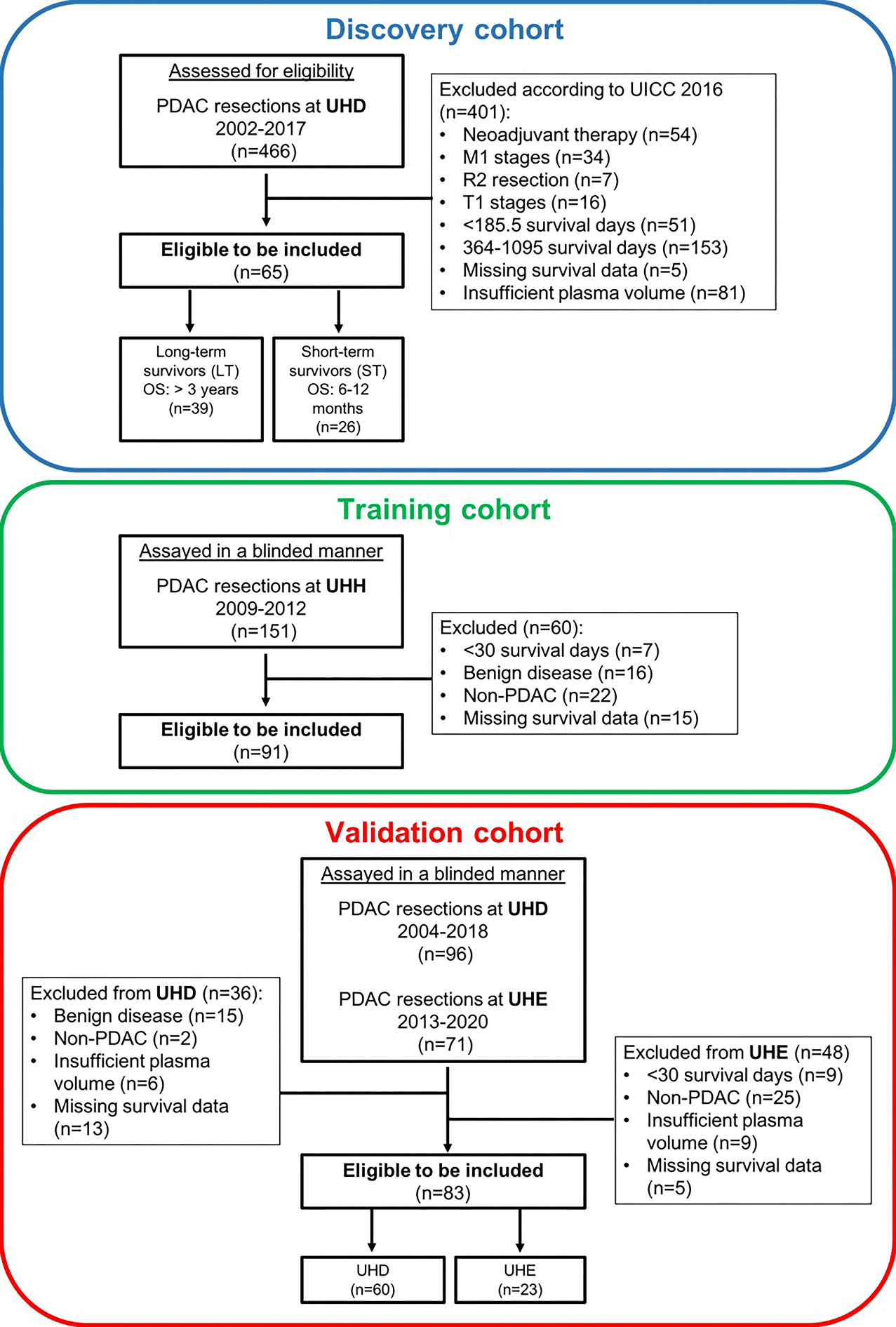


**Fig S1**: Schematic flow chart of the selection of patients in discovery, training and validation cohorts in this study. The number of patients and exclusion criteria for all the cohorts in this study were clearly indicated in the diagram.


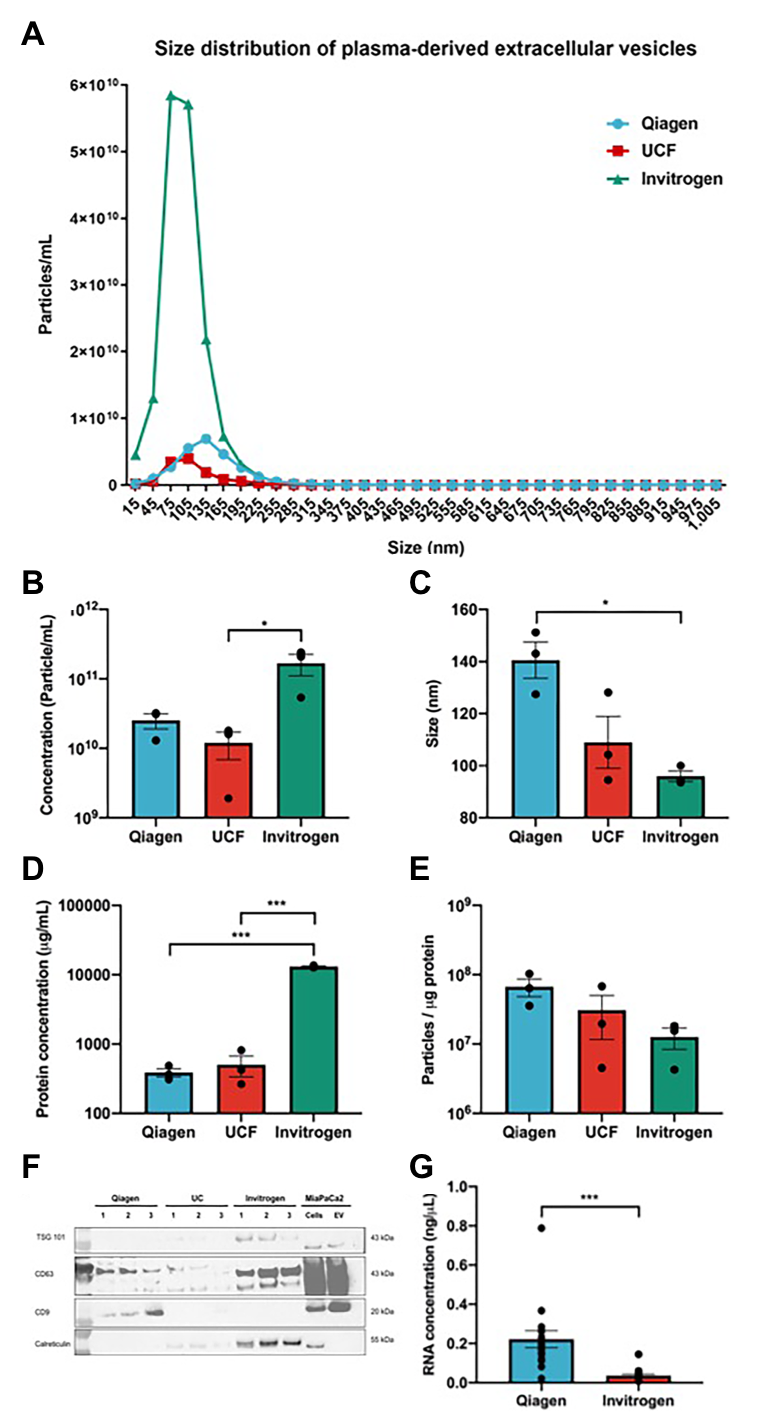


**Fig S2**: Comparison of different EV isolation methods: column-based membrane affinity (exoRNeasy Midi kit from Qiagen company), ultracentrifugation and precipitation (Total Exosome Isolation kit from Invitrogen company). **A-C** Size distribution and nanoparticle tracking analysis (NTA) analysis of the particles from PDAC patients isolated by the abovementioned three methods. Statistical differences were analysed by one-way ANOVA. Data represent the mean + S.E.M. of three patients. **D** Protein concentration of the isolated EVs from PDAC patients quantified by bicinchoninic acid assay (BCA) assay. Statistical differences were analysed by one-way ANOVA. Data represent the mean + S.E.M. of three patients. **E** Purity of EVs determined by the ratio of number of particles and protein concentration, µg. **F** Western blot analysis of the comparison of EV and non-EV protein levels in EVs from PDAC patients isolated by the three different methods. Whole cell lysates and EVs from MiaPaCa2 cell line were used as positive control for this western blot. **G** EV RNA concentration between isolation by Qiagen and Invitrogen kits. Statistical differences were analysed by Mann-Whitney U test. Data represent the mean + S.E.M. of 16 patients.


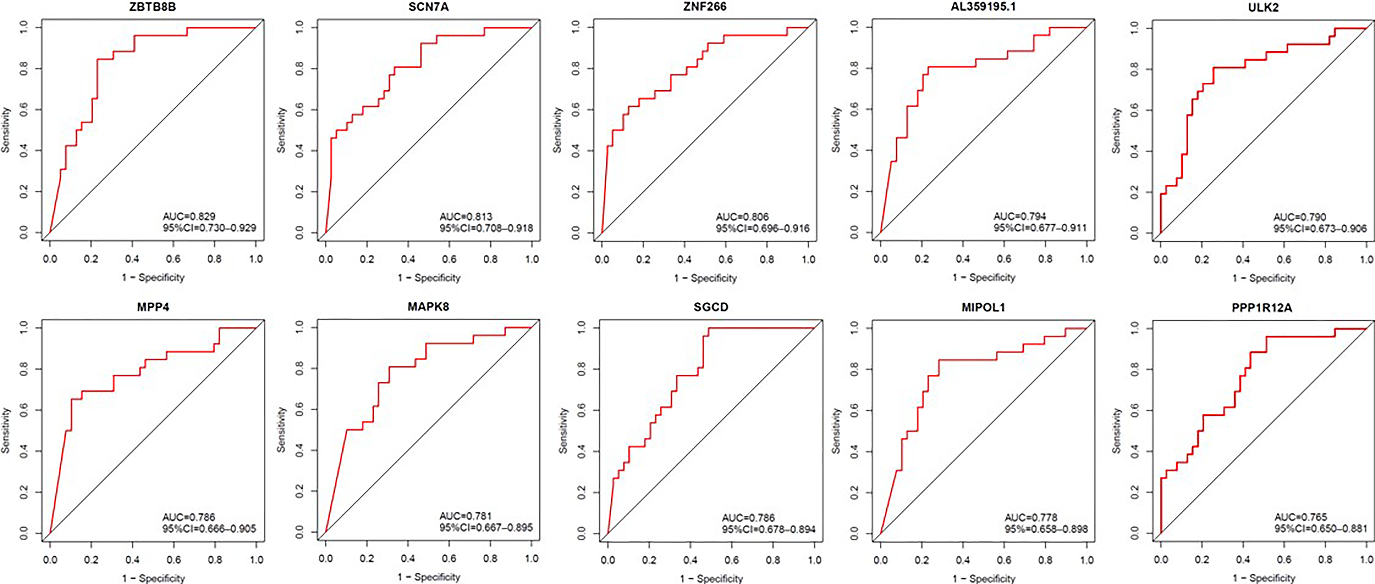


**Fig. S3:** Top-ranked EV mRNAs based on area under the curve (AUC) values from analysis of receiver operating characteristic (ROC) curve. *ZBTB8B*: Zinc Finger And BTB Domain Containing 8B; *SCN7A*: Sodium Voltage-Gated Channel Alpha Subunit 7; *ZNF266*: Zinc Finger Protein 266; *AL359195.1*: Uncharacterized gene; *ULK2*: Unc-51 Like Autophagy Activating Kinase 2, *MPP4*: Membrane Palmitoylated Protein 4, *MAPK8*: Mitogen-Activated Protein Kinase 8; *SGCD*: Sarcoglycan Delta; *MIPOL1*: Mirror-Image Polydactyly 1; *PPP1R12A*: Protein Phosphatase 1 Regulatory Subunit 12A


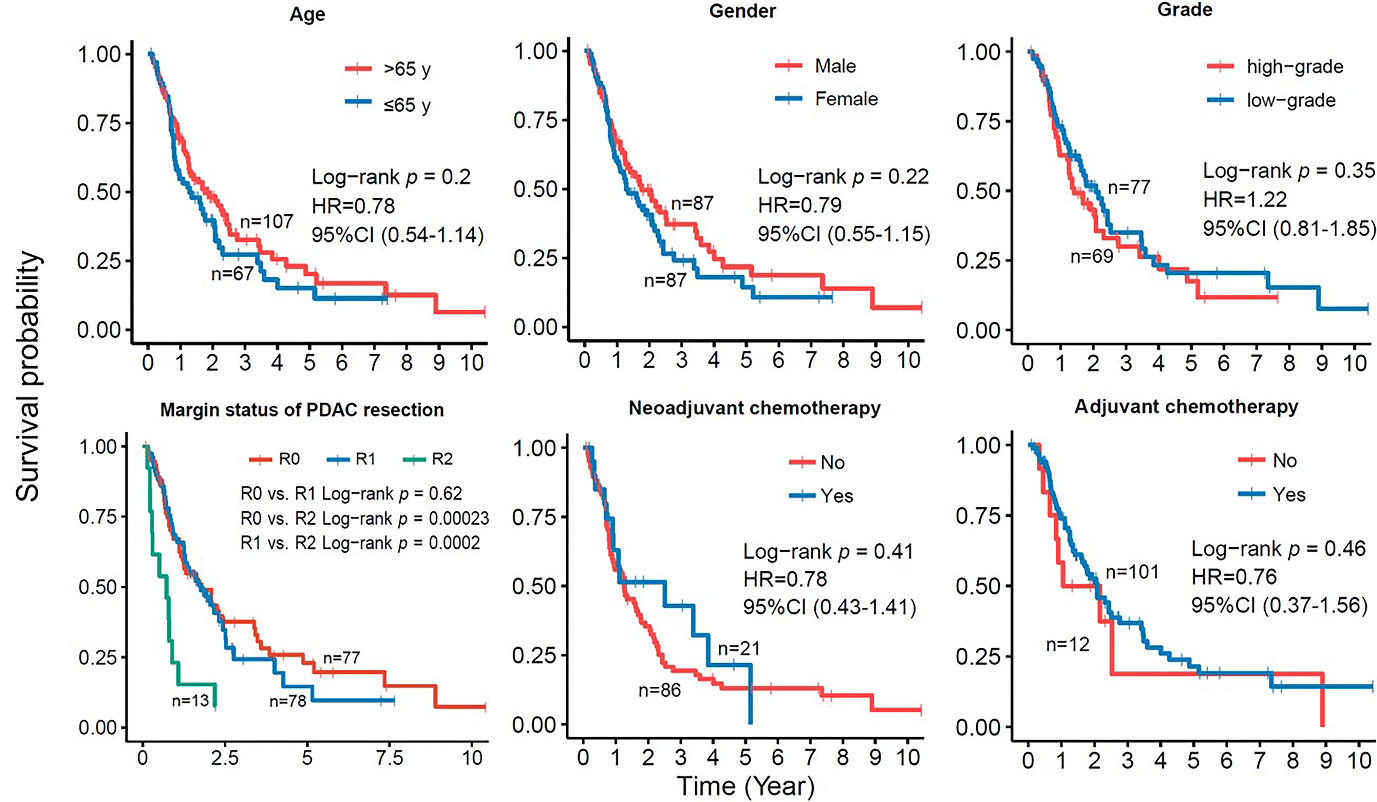


**Fig. S4:** Clinical factors for prediction of overall survival (OS) of the PDAC patients in the combined cohort (training and multi-center validation). The analyses were performed by Kaplan-Meier curves.


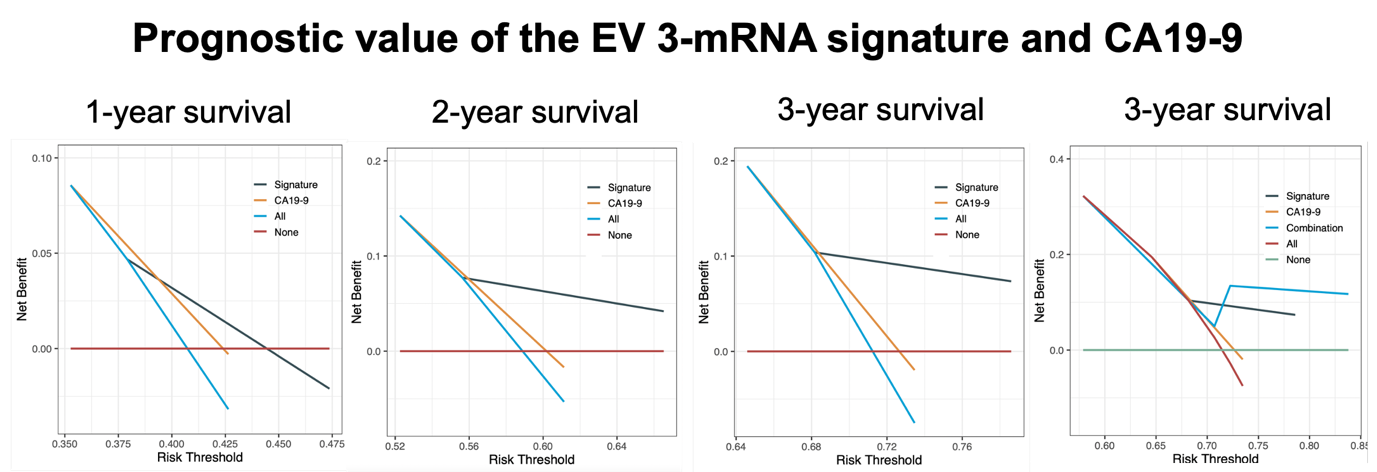


**Fig. S5:** Decision curve analysis to compared the net benefit of EV 3-mRNA signature (dark blue line), CA19-9 (yellow line) or combined EV signature and CA19-9 (blue line in the fourth diagram) for PDAC patients in the combined cohort (training and multi-center validation).

**Table S1:** A summary of the results for EV isolation method comparison.

| **Extracellular vesicle isolation methods** |  | **Extracellular vesicle yield (particle/mL) & size (nm)** |  | **Extracellular vesicle protein concentration**  **(μg/mL)** |  | **Particle ratio / protein concentration** |  | **RNA concentration**  **(ng/μL)** |
| --- | --- | --- | --- | --- | --- | --- | --- | --- |
|  |  |  |  |  |  |  |  |  |
| Membrane affinity (i.e. exoRNeasy Serum/Plasma Midi kit) |  | 2.53 x10^11^ + 6.17 x10^10^ & 140.50 + 6.98 |  | 0.38 + 0.05 |  | 6.75 x10^7^ + 1.95 x10^7^ |  | 0.22 + 0.04 |
| Differential ultracentrifugation |  | 1.20 x10^10^ + 5.07 x10^10^ & 109.00 + 10.02 |  | 0.50 + 0.16 |  | 3.07 x10^7^ + 1.92 x10^7^ |  | - |
| Precipitation (i.e. Total Exosome Isolation kit) |  | 1.68 x10^11^ + 5.77 x10^10^ & 95.93 + 2.04 |  | 13.14 + 0.27 |  | 1.27 x10^7^ + 4.28 x10^7^ |  | 0.04 + 0.01 |
|  |  |  |  |  |  |  |  |  |

**Table S2:** Differentially expressed mRNAs between long-term and short-term PDAC survivors from discovery cohort.

| **Symbol** | **Description** | **Adj. *p*-value** | **Log2FC** |
| --- | --- | --- | --- |
| AL359195.1 | cDNA FLJ46261 fis, clone TESTI4025062 | 0.0334 | -1.2721 |
| BCL2L2-PABPN1 | BCL2L2-PABPN1 readthrough | 0.0015 | 1.7321 |
| BIRC2 | Baculoviral IAP Repeat Containing 2 | 0.0230 | 1.4374 |
| CALD1 | caldesmon 1 | 0.0373 | 1.0449 |
| CCT6A | chaperonin containing TCP1 subunit 6A | 0.0260 | 1.1703 |
| CHST5 | carbohydrate sulfotransferase 5 | 0.0334 | -1.2186 |
| CWC25 | CWC25 spliceosome-associated protein homolog | 0.0015 | 1.5564 |
| EIF5B | eukaryotic translation initiation factor 5B | 0.0014 | 1.5616 |
| GSPT2 | G1 to S phase transition 2 | 0.0430 | 1.2160 |
| HIST1H4H | histone cluster 1, H4h | 0.0495 | 1.6563 |
| LIFR | leukemia inhibitory factor receptor alpha | 0.0468 | -1.0314 |
| LUC7L | LUC7-like | 0.0230 | 1.0966 |
| MAMLD1 | mastermind like domain containing 1 | 0.0055 | 1.3992 |
| MAPK8 | mitogen-activated protein kinase 8 | 0.0334 | -1.2995 |
| MED19 | mediator complex subunit 19 | 0.0230 | 1.3949 |
| MIPOL1 | mirror-image polydactyly 1 | 0.0393 | -1.1220 |
| MPP4 | membrane palmitoylated protein 4 | 0.0351 | -1.2120 |
| MSL2 | male-specific lethal 2 homolog (Drosophila) | 0.0424 | 1.3131 |
| MXD1 | MAX dimerization protein 1 | 0.0230 | 1.3384 |
| NDUFAF3 | NADH dehydrogenase complex I, assembly factor 3 | 0.0432 | 1.0924 |
| PABPN1 | poly(A) binding protein nuclear 1 | 0.0015 | 1.7288 |
| PGBD5 | piggyBac transposable element derived 5 | 0.0468 | -1.0716 |
| PPP1R12A | protein phosphatase 1 regulatory subunit 12A | 0.0012 | 1.5891 |
| RAD21 | RAD21 cohesin complex component | 0.0260 | 1.1388 |
| SCN7A | sodium voltage-gated channel alpha subunit 7 | 0.0413 | -1.0304 |
| SGCD | sarcoglycan delta | 0.0216 | -1.0427 |
| SNN | stannin | 0.0468 | 1.2006 |
| TMEM209 | transmembrane protein 209 | 0.0498 | -1.0956 |
| ULK2 | unc-51 like autophagy activating kinase 2 | 0.0056 | -1.0776 |
| ZBTB8B | zinc finger and BTB domain containing 8B | 0.0015 | -1.2096 |
| ZNF266 | zinc finger protein 266 | 0.0334 | -1.0967 |

Adj. *p*-value: adjusted *p*-value; Log2FC: Log2 fold-change
